# Deep learning for high-resolution seismic imaging

**DOI:** 10.1038/s41598-024-61251-8

**Published:** 2024-05-06

**Authors:** Liyun Ma, Liguo Han, Qiang Feng

**Affiliations:** Jilin University, College of Geoexploration Science and Technology, Changchun, 130026 China

**Keywords:** Solid Earth sciences, Engineering

## Abstract

Seismic imaging techniques play a crucial role in interpreting subsurface geological structures by analyzing the propagation and reflection of seismic waves. However, traditional methods face challenges in achieving high resolution due to theoretical constraints and computational costs. Leveraging recent advancements in deep learning, this study introduces a neural network framework that integrates Transformer and Convolutional Neural Network (CNN) architectures, enhanced through Adaptive Spatial Feature Fusion (ASFF), to achieve high-resolution seismic imaging. Our approach directly maps seismic data to reflection models, eliminating the need for post-processing low-resolution results. Through extensive numerical experiments, we demonstrate the outstanding ability of this method to accurately infer subsurface structures. Evaluation metrics including Root Mean Square Error (RMSE), Correlation Coefficient (CC), and Structural Similarity Index (SSIM) emphasize the model's capacity to faithfully reconstruct subsurface features. Furthermore, noise injection experiments showcase the reliability of this efficient seismic imaging method, further underscoring the potential of deep learning in seismic imaging.

## Introduction

Seismic imaging technology, as a method to obtain information about underground geological structures by analyzing the propagation and reflection of seismic waves, plays a crucial role in geological exploration^1–3^, resource development^4–6^, underground water detection^7,8^, and other fields. This technology records the vibration signals of seismic waves propagating underground using seismic instruments and processes and interprets these data through mathematical algorithms and signal processing techniques to obtain the physical properties and structural characteristics of underground media. The imaging goal is to visualize underground layer boundaries, structural features, etc., to assist geologists in further analyzing and interpreting underground structures.

Despite the critical position of seismic imaging technology in geological exploration and resource development, traditional methods have theoretical and practical limitations in resolution. These limitations stem from theoretical constraints in underground wave propagation physics and the computational costs associated with high-frequency imaging. Resolution limitations make it difficult to accurately depict details of underground structures, especially in areas with geological complexity and media heterogeneity. In recent years, the increasing demand for seismic exploration accuracy has driven researchers to improve the resolution of seismic images through various means^9^. For example, high-density acquisition can enhance horizontal resolution, while recording wide-band seismic data can improve vertical resolution^10,11^.

The rise of deep learning technology has opened up new possibilities in the field of seismic exploration^12–19^, encompassing various aspects such as data processing, imaging, and inversion. The application of deep learning in seismic imaging mainly focuses on processing seismic images, improving the quality of seismic imaging results by establishing mappings between low-resolution and high-resolution versions^20,21^. Specifically, researchers have used deep learning techniques to compensate for absorption and correct dispersion, achieving the goal of accelerating high-resolution seismic imaging^22^. Additionally, some studies have introduced structural constraints into neural network frameworks to perform seismic high-resolution reconstruction, achieving significant results^23^.

However, the aforementioned high-resolution imaging methods mainly focus on post-processing of low-resolution results. In contrast, our research endeavors to achieve high-resolution imaging directly through the synergistic combination of Transformer and Convolutional Neural Network (CNN). The network takes seismic data as input and the desired output is reflection model. This integration capitalizes on the strengths of both models: Transformers excel in capturing long-range dependencies and global contextual information^24^, while CNNs are adept at capturing local spatial features and patterns. By harnessing the complementary capabilities of Transformer and CNN, our proposed neural network architecture aims to enhance the resolution and fidelity of seismic imaging. Additionally, the incorporation of the Adaptively Spatial Feature Fusion (ASFF) method further enriches the model's ability to extract and integrate spatial features adaptively, contributing to the accurate mapping of seismic data onto reflection models.

## Methods

### Review of seismic imaging

The goal of seismic imaging is to infer subsurface structures based on observed seismic data. This can be achieved by solving inverse problems. Reverse Time Migration (RTM) is an imaging technique based on the wave equation^25^, which utilizes the cross-correlation of the underground forward and backward wavefields, demonstrating excellent adaptability, especially in areas with complex structures and high velocity variations. The formula for the cross-correlation imaging condition is expressed as:

1 $$I(x,z)={\int }_{0}^{T}{u}_{\text{f }}(x,z,t)*{u}_{\text{b }}(x,z,t)dt$$

Here, $I(x,z)$ represents the RTM result, ${u}_{\text{f }}(x,z,t)$ denotes the forward wavefield, and ${u}_{\text{b }}(x,z,t)$ is the backward wavefield.

However, RTM suffers from low-frequency noise and inaccurate amplitudes, limiting its application in seismic imaging. To address the shortcomings of RTM, Least Squares Reverse Time Migration (LSRTM) associates the migration imaging result with seismic data^26^, constructing the least squares objective function:

2 $$E({\varvec{m}})=\frac{1}{2}{\parallel {\varvec{L}}{\varvec{m}}-{{\varvec{d}}}_{{\text{obs}}}\parallel }^{2}$$

Here, ${{\varvec{d}}}_{{\text{obs}}}$ represents the observed data, ${\varvec{L}}$ is the forward operator, and ${\varvec{m}}$ is the subsurface structural parameter.

LSRTM involves key steps such as forward simulation, backpropagation, gradient computation, and optimization algorithms. Through iterative optimization to minimize the error between observed and simulated data, LSRTM enhances the quality of seismic imaging.

### Deep neural network for seismic imaging

**Figure 1:**
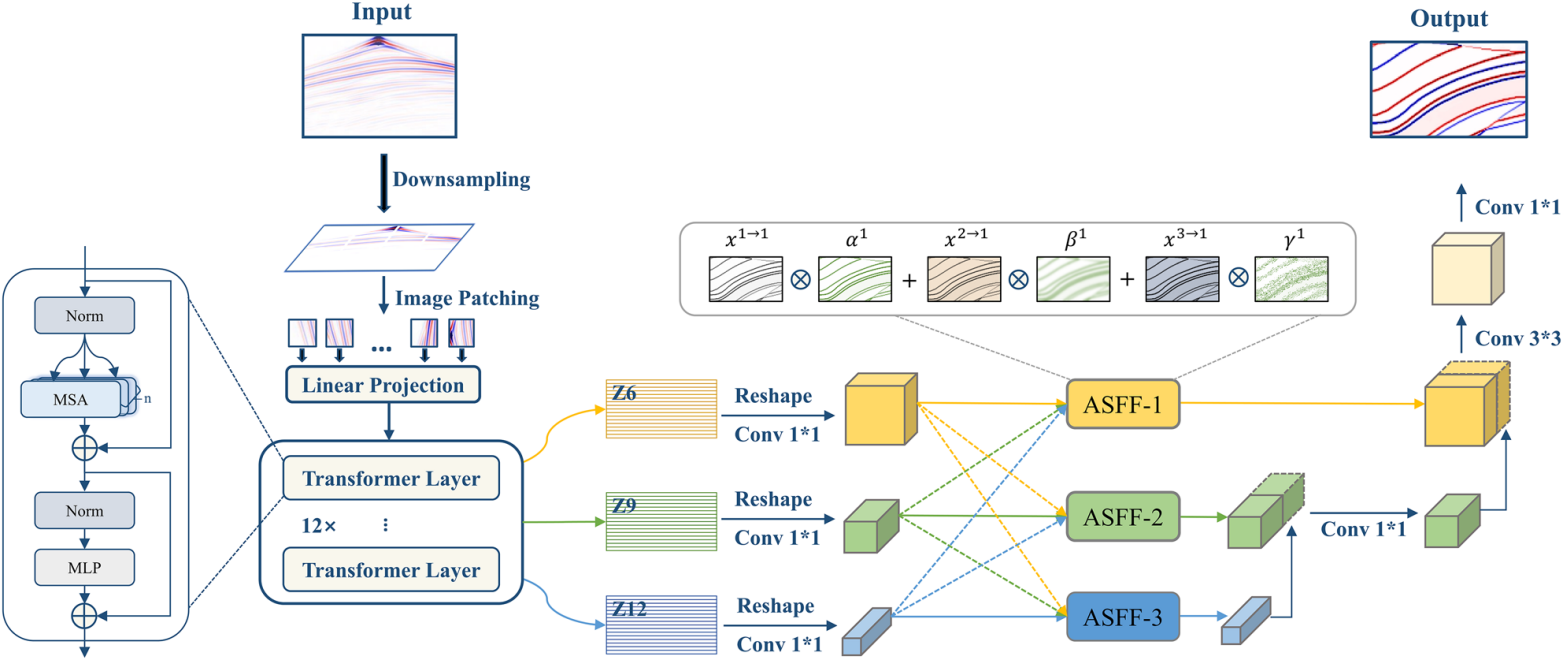
Network architecture diagram.

In this study, we introduce a hybrid architecture (Fig. 1) that integrates Transformer and CNN to address seismic imaging tasks. Within the Transformer framework, the need for a one-dimensional sequence as input necessitates an initial transformation of the input image. The Image Patching phase involves partitioning the input image into a series of equally sized image patches, each with a size of ${P}^{2}$. This transforms the original $H\times W$ image into an $N\times P\times P$ sequence, where $N$ represents the sequence length, encompassing $\frac{H\times W}{{P}^{2}}$ image patches. Consequently, the input image is reshaped into a one-dimensional sequence, with each image patch corresponding to a vector. The adoption of a smaller patch size enables enhanced capture of intricate details within the image, thus elevating the model's accuracy, albeit at the expense of increased computational overhead^27^. In view of balancing between model efficacy and computational efficiency, we establish $P=16$. In the Input Embedding stage, a linear transformation is applied to each segmented image patch, mapping it to a continuous vector representation. As the Transformer model abstains from utilizing recurrent or convolutional layers for sequence processing, positional encoding is incorporated into the input embedding vector to discern the positional information of each image patch.

3 $${\mathbf{Z}}_{0}=[{{\varvec{X}}}_{p}^{1}\mathbf{E};{\mathbf{X}}_{p}^{2}\mathbf{E};\dots ;{\mathbf{X}}_{p}^{N}\mathbf{E}]+{\mathbf{E}}_{\text{pos}}$$

The proposed model employs a Transformer Encoder comprising ${\text{L}}=12$ layers to process the image sequence, with each encoder layer composed of Multi-Head Self-Attention (MSA) and Multi-Layer Perceptron (MLP).

4 $${\mathbf{Z}}_{l}^{\mathrm{^{\prime}}}={\text{MSA}}({\text{LN}}({\mathbf{Z}}_{l-1}))+{\mathbf{Z}}_{l-1},l=1\dots L$$

5 $${\mathbf{Z}}_{l}={\text{MLP}}({\text{LN}}({\mathbf{Z}}_{l}^{\mathrm{^{\prime}}}))+{\mathbf{Z}}_{l}^{\mathrm{^{\prime}}},l=1\dots L$$

Here, ${\text{LN}}(\cdot )$ denotes layer normalization, $l$ is the identifier for intermediate blocks, and *L* is the number of Transformer layers.

These stacked Transformer layers facilitate capturing the complexity of the data from a multiscale perspective. To prevent the loss of primary features by solely relying on the last layer output, we employ a multi-level feature extraction strategy. In addition to the final layer (12th layer), features are extracted from the 6th and 9th layers, representing deep, intermediate, and shallow features, providing a rich and multiscale feature space. These three layers of features are adjusted to different resolutions of feature maps and fused through ASFF, resulting in adaptive aggregation at each scale.

ASFF constitutes an attention-based spatial feature integration strategy devised to amalgamate feature maps originating from diverse spatial resolutions within deep neural networks^28^. Its principal objective is to augment the model's perceptual acuity concerning targets across varying scales. ASFF dynamically weights and fuses features from distinct spatial resolutions by learning task-specific attention weights.

We represent features at resolution level ${\ell}$ (where ${\ell}\in \left\{\mathrm{1,2},3\right\}$) as ${x}^{l}$. For level ${\ell}$, we resize features from other levels $n$ ($n\ne {\ell}$) to the same shape as ${x}^{l}$. Let ${x}_{ij}^{n\to {\ell}}$ denote the feature vector at position $(i,j)$ on the feature map, adjusted from level $n$ to level ${\ell}$. We perform the following fusion of corresponding level ${\ell}$ features:

6 $${y}_{ij}^{{\ell}}={\alpha }_{ij}^{{\ell}}\cdot {x}_{ij}^{1\to {\ell}}+{\beta }_{ij}^{{\ell}}\cdot {x}_{ij}^{2\to {\ell}}+{\gamma }_{ij}^{{\ell}}\cdot {x}_{ij}^{3\to {\ell}}$$

Here, ${y}_{ij}^{{\ell}}$ signifies the vector at position $(i,j)$ in the output feature map ${y}^{{\ell}}$ across channels. The spatial importance weights ${\alpha }_{ij}^{{\ell}}$, ${\beta }_{ij}^{{\ell}}$, and ${\gamma }_{ij}^{{\ell}}$ for features from three different levels to level ${\ell}$ are adaptively learned by the network. To ensure the effectiveness of weights, constraints ${\alpha }_{ij}^{{\ell}}+{\beta }_{ij}^{{\ell}}+{\gamma }_{ij}^{{\ell}}=1$ and ${\alpha }_{ij}^{{\ell}}, {\beta }_{ij}^{{\ell}}, {\gamma }_{ij}^{{\ell}}\in [\mathrm{0,1}]$ are enforced. These constraints ensure the validity and range of the weights. The weights are computed using softmax functions with control parameters as follows:

7 $${\alpha }_{ij}^{{\ell}}=\frac{{e}^{{\lambda }_{{\alpha }_{ij}}^{{\ell}}}}{{e}^{{\lambda }_{{\alpha }_{ij}}^{{\ell}}}+{e}^{{\lambda }_{{\beta }_{ij}}^{{\ell}}}+{e}^{{\lambda }_{{\gamma }_{ij}}^{{\ell}}}}$$

8 $${\beta }_{ij}^{{\ell}}=\frac{{e}^{{\lambda }_{{\beta }_{ij}}^{{\ell}}}}{{e}^{{\lambda }_{{\alpha }_{ij}}^{{\ell}}}+{e}^{{\lambda }_{{\beta }_{ij}}^{{\ell}}}+{e}^{{\lambda }_{{\gamma }_{ij}}^{{\ell}}}}$$

9 $${\gamma }_{ij}^{{\ell}}=\frac{{e}^{{\lambda }_{{\gamma }_{ij}}^{{\ell}}}}{{e}^{{\lambda }_{{\alpha }_{ij}}^{{\ell}}}+{e}^{{\lambda }_{{\beta }_{ij}}^{{\ell}}}+{e}^{{\lambda }_{{\gamma }_{ij}}^{{\ell}}}}$$

The calculation of control parameters ${\lambda }_{{\alpha }_{ij}}^{{\ell}}$,${\lambda }_{{\beta }_{ij}}^{{\ell}}$, and ${\lambda }_{{\gamma }_{ij}}^{{\ell}}$ is performed through $1{\text{x}}1$ convolution layers from ${x}_{ij}^{1\to {\ell}}$,${x}_{ij}^{2\to {\ell}}$, and ${x}_{ij}^{3\to {\ell}}$, respectively. These parameters are learned through standard backpropagation during network training.

Overall, this approach furnishes the model with a rich and multiscale feature space, thereby contributing to its performance in complex seismic imaging tasks.

## Results

### Dataset and training

**Figure 2:**
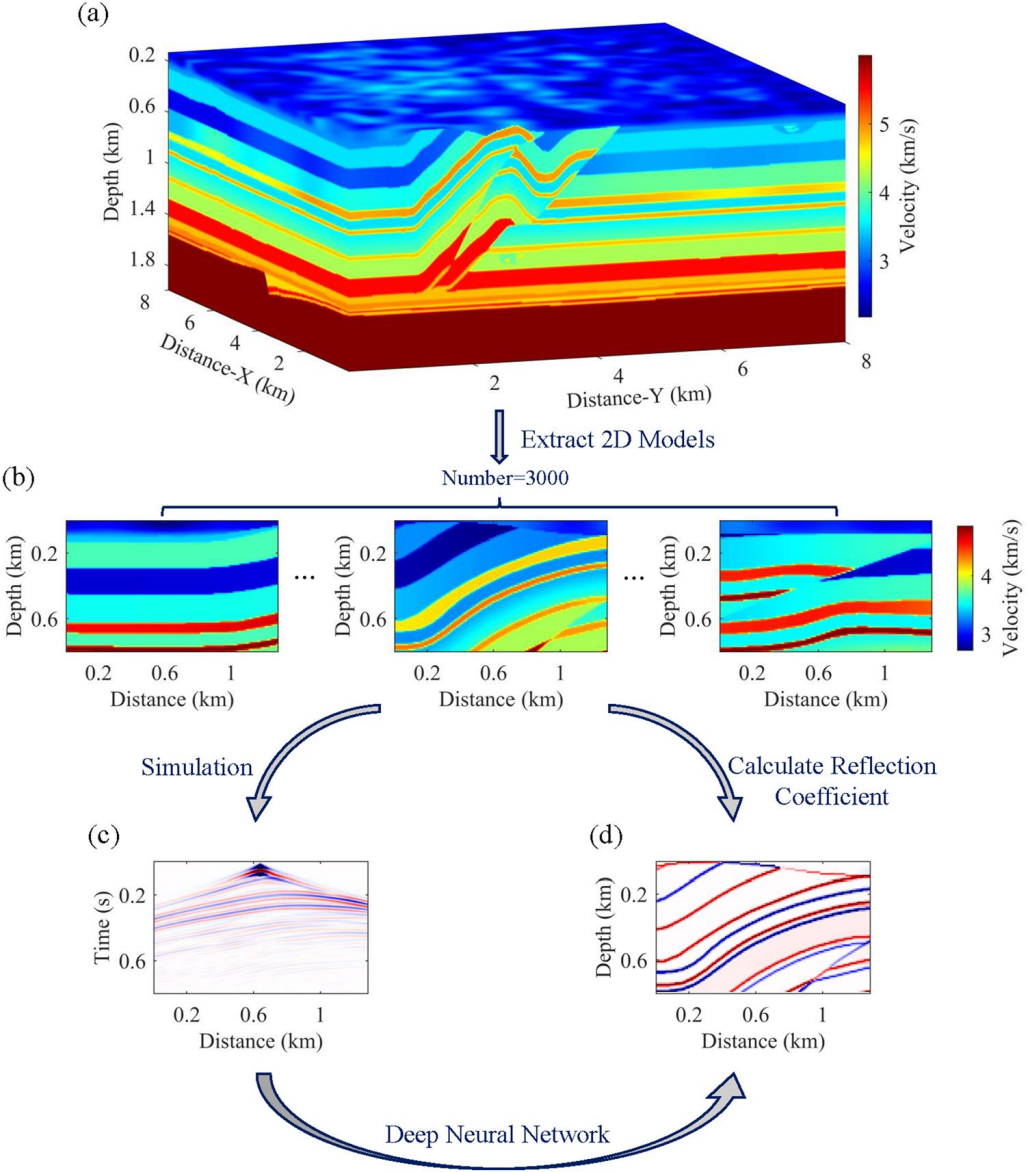
Schematic of research procedures. (**a**) 3D overthrust model. (**b**) 2-D velocity models extracted from (**a**). (c) Seismic record, representing input data. (**d**) Reflectivity model, representing label data.

The research process is clearly shown in Fig. 2. The dataset utilized in this study derives from the 3-D Overthrust Model (Fig. 2a). Synthetic data is generated through simulations on multiple diverse 2-D models (Fig. 2b) extracted from the 3-D Model. Finite-difference modeling is employed to solve the 2-D acoustic wave equation in the time domain, with a Ricker wavelet of 30 Hz serving as the source signal. Three perfectly matched layers (PML) are placed respectively at the bottom, left, and right sides of the model, with free boundary conditions applied at the models' top to produce seismic records as input data (Fig. 2c). By calculating velocity differences between adjacent layers and subsequently deriving reflection coefficients based on these disparities, a multitude of reflection models serve as labeled data (Fig. 2d). The deep neural network is trained to establish a direct mapping from the seismic record to the reflection model.

**Figure 3:**
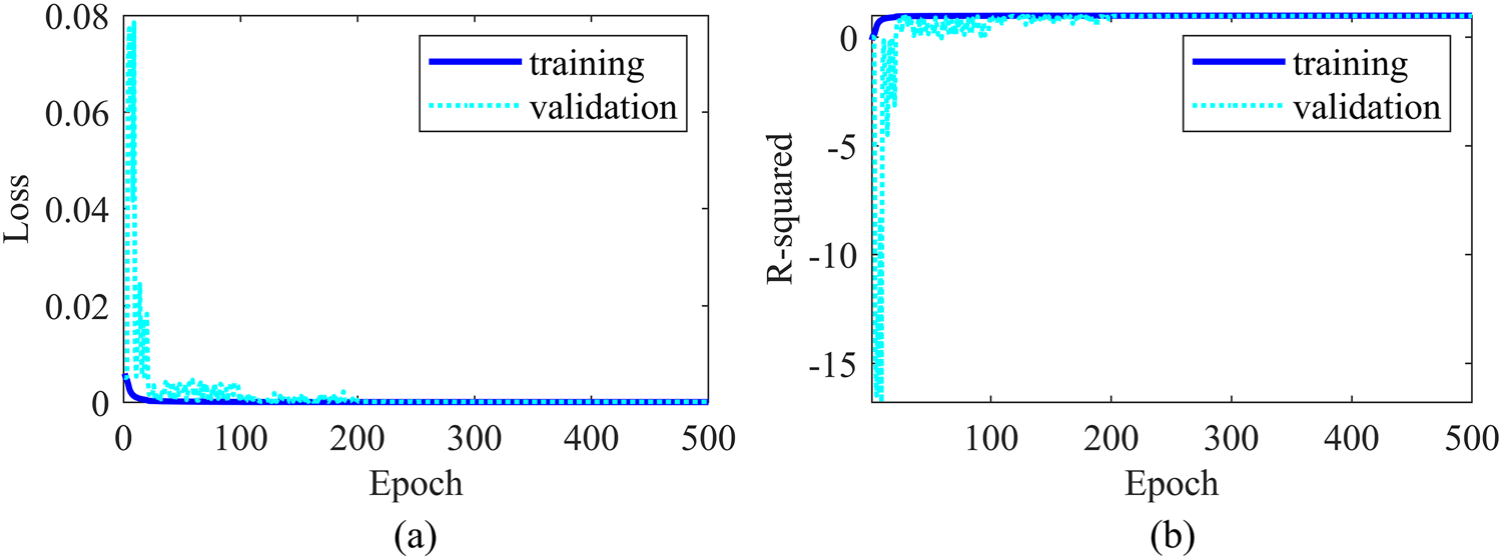
(**a**) Loss curves and (**b**) evaluation metrics for training and validation sets across 500 epochs.

The dataset was partitioned into training, validation, and test sets in an 8:1:1 ratio. During the training process, we employed the ADAM optimizer with a batch size of 32, and the learning rate decayed with iterations. Mean Squared Error (MSE) was utilized as the loss function to measure the disparity between predicted outcomes and the true reflectivity models. By minimizing MSE, we facilitated the neural network in learning a more precise mapping relationship from seismic records to reflectivity models. To evaluate the model's performance, R-squared was chosen as the primary assessment metric. R-squared, a statistical indicator ranging from 0 to 1, quantifies the goodness of fit of the model to the data. A value closer to 1 indicates a stronger explanatory power of the model. Our objective was to maximize R-squared, ensuring the model accurately predicts reflectivity models corresponding to seismic records. The loss curves and evaluation metrics of the training and validation sets over 500 epochs are presented in Fig. 3.

### Test results

**Figure 4:**
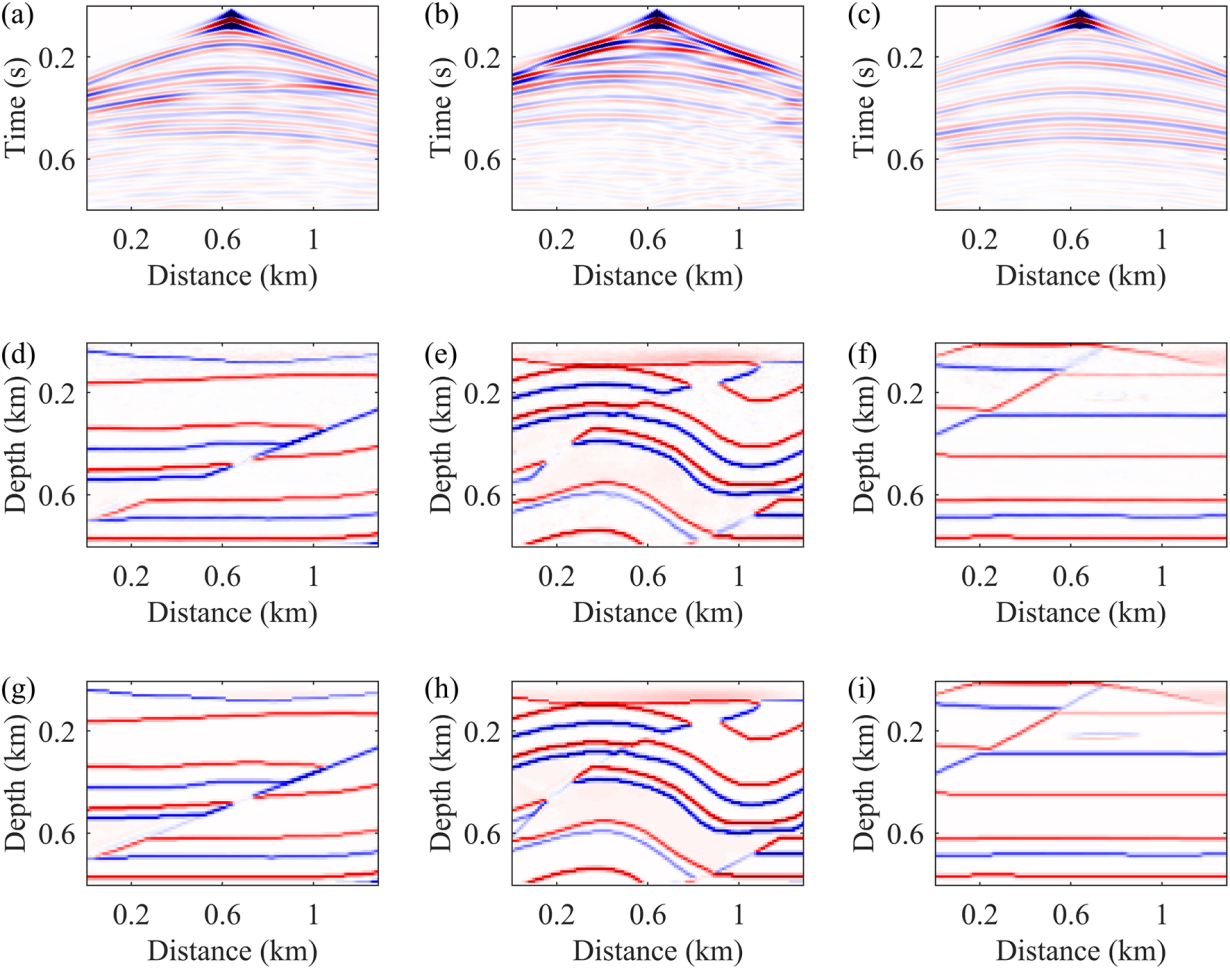
Three sets of test data, each including input data (**a**–**c**), neural network-generated reflectivity models (**d**–**f**), and ground truth reflectivity models (**g**–**i**).

**Figure 5:**
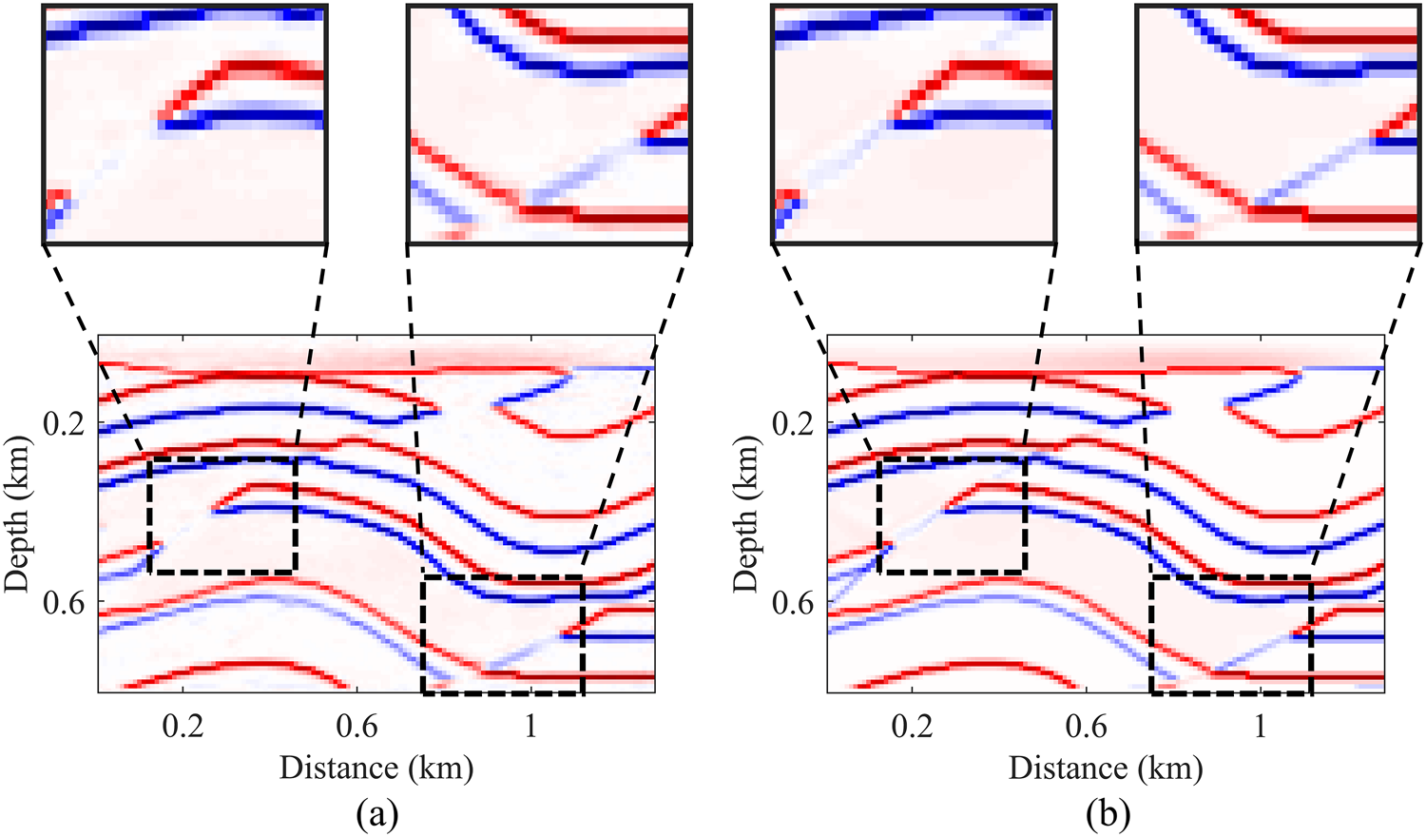
Magnified comparison between (**a**) predicted result and (**b**) true reflectivity model. Black dashed-line rectangles indicate areas to be magnified, while black solid-line rectangles represent the magnified regions.

Upon completion of neural network training, we conduct a comprehensive analysis of the training results to evaluate the method's ability to accurately infer underground structures from seismic records. The data types used in the testing are synthetic data. Initially, three sets of test data are selected for comparison, each comprising input data, neural network-generated reflectivity models, and ground truth reflectivity models (Fig. 4). We observe excellent performance of the neural network model in high-amplitude (strong reflection) areas, demonstrating its effectiveness in capturing key geological features and exhibiting consistent morphology with the real model. Subsequently, a subset of test results is chosen for detailed comparison (Fig. 5). We magnify local regions of predicted results and actual reflectivity models for detailed comparison. Amplification of imaging results in weak reflection areas, indicated by black rectangular boxes, shows the model's effectiveness in reproducing small-scale geological features. However, performance in extremely weak reflection areas requires further enhancement.

To quantify the predictive performance of the model, we employ the following metrics for evaluation: Root Mean Square Error (RMSE), Correlation Coefficient (CC), and Structural Similarity Index (SSIM). RMSE is widely used to assess model prediction errors, measuring the square root of the mean squared differences between predicted and observed values. CC evaluates the linear correlation between model predictions and actual observations, with values ranging from -1 to 1, where values closer to 1 indicate stronger linear relationships between predictions and observations. SSIM, a measure of structural similarity, compares the similarity between two images, considering differences in brightness, contrast, and structure. A comprehensive summary of the evaluation results is provided in Table 1. The aforementioned comparisons collectively reflect the ability of the proposed method to accurately infer underground structures from seismic records.

**Table 1:** Comparison of evaluation metrics between test results and label data.

Comparative scope	SSIM	CC	RMSE
Single	0.9954	0.9929	0.0024
Average	0.9831	0.9914	0.0189

**Figure 6:**
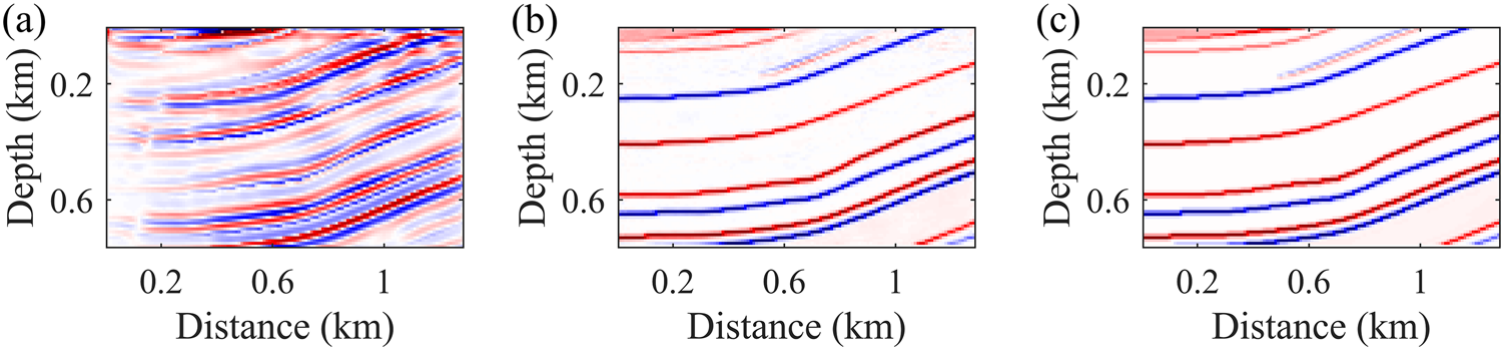
Comparison of imaging results. (**a**) Result of RTM. (**b**) Result of neural network. (**c**) Real reflectivity model.

We conduct a comparative analysis between the proposed method and the conventional RTM approach, with the results displayed in Fig. 6. The subplots (a), (b), and (c) respectively depict the RTM imaging result, the neural network's prediction result, and the actual reflectivity model used as a reference. It is observed that this method significantly improves imaging accuracy and resolution. Through end-to-end processing using deep learning model, we achieve better capture of subtle underground structural features, thereby achieving higher resolution in seismic imaging.

### Noise injection experiment

In seismic data processing, Gaussian noise is regarded as a significant interference factor characterized by its ubiquitous presence in the natural environment and its probability density function following a normal distribution. To more accurately simulate the observational conditions of seismic data in the real world, Gaussian noise is incorporated into the dataset. This process entails generating random noise conforming to a Gaussian distribution and adding it to the original dataset. The signal-to-noise ratio (SNR) of the added noise is randomly distributed between 5 to 15 decibels, as per the following formula:

10 $$SNR=10{{\text{log}}}_{10}\frac{{P}_{s}}{{P}_{n}}$$

Here, ${P}_{s}$ refers to the power of the effective signal, while ${P}_{n}$ refers to the power of the noise.

**Figure 7:**
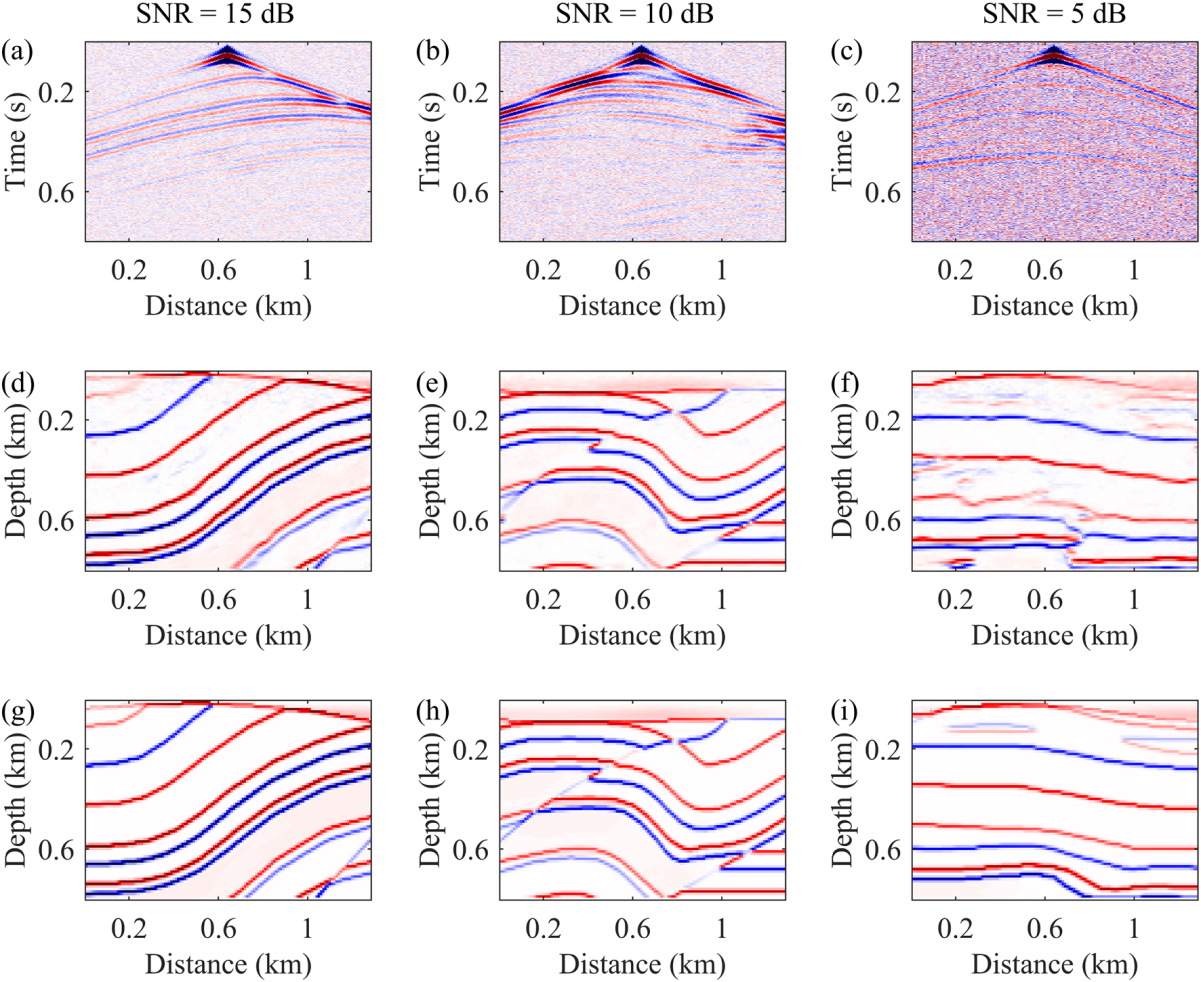
Three sets of test data, each including input data with noise (**a**–**c**), neural network-generated reflectivity models (**d**–**f**), and ground truth reflectivity models (**g**–**i**).

Figure 7 illustrates three sets of test data, each comprising input data with added noise (a–c), reflectivity models generated by the neural network (d–f), and ground truth reflectivity models (g–i). The presence of Gaussian noise significantly obscures the effective reflection signals in seismic data, particularly making phase identification extremely challenging under higher noise levels. Despite the presence of noise, the reflectivity models generated by the neural network still accurately capture underground structural features to a certain extent, especially demonstrating more reliable performance at lower noise levels. This suggests that the proposed seismic imaging method exhibits a degree of noise tolerance and remains effective in revealing underground structural features across varying noise levels.

## Discussion

The proposed deep learning model delineates a direct mapping from seismic recordings to subsurface reflectivity models, thereby circumventing the intermediate step of velocity inversion typically associated with inverse problems in seismic exploration. This approach directly addresses the fundamental objective of characterizing subsurface reflectivity, thereby potentially simplifying the seismic imaging process and facilitating a deeper understanding of subsurface structures with heightened precision and efficiency.

Looking forward, the trajectory of future improvements is poised towards fortifying seismic imaging methodologies to accommodate heightened levels of noise within datasets. As technological advancements continue to push the boundaries of data acquisition, seismic datasets are increasingly susceptible to noise from various sources, including environmental factors and instrumentation limitations. Addressing this challenge is crucial for ensuring the reliability and accuracy of seismic imaging across diverse geological contexts.

However, practical application of the proposed method to real seismic datasets remains a challenge. Deployment in field scenarios may require utilizing extensive synthetic datasets for training. Subsequent work will focus on enhancing the model's ability to generalize from synthetic to field data, representing a key direction for our future research efforts.

Furthermore, employing deep learning techniques for seismic data processing inevitably confronts inherent limitations and challenges associated with the technology. It necessitates ample training data for achieving optimal performance, and predictions may exhibit unreliability in scenarios where training datasets are insufficient. Moreover, the interpretability of neural network models in terms of their learning and prediction mechanisms remains elusive.

## Conclusion

In this study, we propose a seismic imaging approach based on deep learning. Specifically, we establish a neural network that combines Transformer and CNN architectures, augmented with the ASFF modules, enabling direct transformation of seismic data into underground reflectivity models. Numerical experiments demonstrate the efficacy of the approach in enhancing seismic imaging resolution. Additionally, the robustness of the model to noise is demonstrated.

## Data Availability

The datasets generated during and analysed during the current study are available from the corresponding author on reasonable request.
